# Reviewed article: Junior CAR, Santos SS, Amaral TLM. Trend and burden of lower-limb amputations due to diabetes: time series analysis, Amazonas, 2012-2021. Epidemiol Serv Saude. 2026:35;e20250534

**DOI:** 10.1590/S2237-96222026v35e20250534.a

**Published:** 2026-03-16

**Authors:** Luciene Fátima Fernandes Almeida

**Affiliations:** 1 Fundação Oswaldo Cruz, Rio de Janeiro, RJ, Brazil Rio de Janeiro RJ Brazil

## Peer review A

## Questionnaire

Does the manuscript contain new and significant information to justify publication? Yes

Does the Abstract (Summary) clearly and accurately describe the content of the article? Yes

Is the problem significant and concisely stated? Yes

Are the methods described comprehensively? Yes

Are the interpretations and conclusions justified by the results? Yes

Is adequate reference made to other work in the field? Yes

## Manuscript Structure

Length of article is: Adequate

Number of tables is: Adequate

Number of figures is: Adequate

Please state any conflict(s) of interest that you have in relation to the review of this paper. None

Interest: Average

Quality: Good

Originality: Average

Overall: Average

Recommendation: Minor Revision

## Comments

Prezados autores,

Agradeço a oportunidade de avaliar o manuscrito intitulado “Tendência e carga de amputações de membros inferiores por diabetes no Amazonas, Brasil: análise de série temporal, 2012-2021”. Trata-se de um estudo ecológico exploratório de séries temporais com o objetivo de estimar a tendência temporal e a carga da doença para as amputações de membros inferiores atribuíveis ao diabetes da população geral do estado do Amazonas, no período de 2012 a 2021.

Os autores conduzem o estudo com uma base amplamente utilizada em estudos epidemiológicos, o SIH/SUS. O estudo apresenta objetivo claro e bem definido. O delineamento escolhido e os métodos foram apropriados para responder à pergunta de pesquisa. Tabelas foram bem elaboradas e são de fácil compreensão. O resumo está bem estruturado e apresenta com clareza o objetivo, métodos, resultados e conclusão do trabalho. Na seção de discussão, os autores, mesmo que com limitações, discutem adequadamente os resultados à luz da literatura e indicam a relevância dos achados como subsídio para o desenvolvimento de ações de promoção da saúde e controle do diabetes na população brasileira. Além disso, as conclusões do estudo são sustentadas pelos resultados.

No entanto, faço alguns comentários, maiores e menores, necessários ao aprimoramento da redação do manuscrito.

Comentários maiores:

Comentários para a seção de Introdução:

Nesta seção, é necessário aprimorar a fundamentação teórica que destaca a originalidade do estudo e, concomitantemente, justifica a importância de que essa investigação seja conduzida no estado do Amazonas. Os autores apresentam dados epidemiológicos sobre a temática no referido estado, mas não contextualizam como este cenário se diferencia do restante do Brasil.

Da mesma forma, é necessário que os autores forneçam embasamento teórico que justifique que as análises sejam conduzidas considerando a estratificação por sexo.

Comentários para a seção de Discussão:

Os autores mencionam que, devido a limitações no preenchimento correto e completo da Autorização de Internação Hospitalar, há possibilidade de viés de informação no estudo. Importante ressaltar que viés de informação resulta de uma tendência sistemática de alocar erroneamente os indivíduos do estudo nas diferentes categorias de exposição ou de desfecho (SZKLO, M.; NIETO, F. J. Epidemiology: beyond the basics. 4. ed. Burlington: Jones & Bartlett Learning, 2019.). Dessa forma, sugiro que os autores reflitam melhor se realmente há possibilidade de viés no presente estudo ou se há limitações da base de dados que influenciam os resultados do estudo. Algumas questões que podem auxiliar nessa compreensão seriam: a classificação das internações poderia ser diferente conforme região do estudo, sexo, idade do paciente e isso influenciaria nas medidas estimadas?

Além disso, outras limitações inerentes ao SIH/SUS precisam ser mais bem discutidas no sentido de indicar suas implicações nos resultados encontrados. Quais as implicações da impossibilidade de avaliação de reinternações hospitalares para os resultados encontrados?

Acredito que a ausência de dados acerca das condições sociais e econômicas dos indivíduos, importantes na determinação do estado de saúde e do uso dos serviços de saúde, também podem ser mencionadas e discutidas enquanto limitação do estudo. Além disso, o SIH/SUS é utilizado para fins de reembolso dos serviços de assistência hospitalar. Quais as implicações dessa característica para os resultados encontrados?

Comentários menores:

Comentários para a seção de Métodos:

Nesta seção, sugiro que os autores justifiquem o recorte temporal para a análise de tendência temporal.

Ao mencionar os critérios para identificar o número total de cirurgias de amputação, sugiro que seja esclarecido se o diagnóstico refere-se ao diagnóstico primário.

A análise de tendência temporal foi conduzida entre os anos de 2012 a 2021. No entanto, os autores mencionam que utilizaram dados de estimativa populacional do IBGE para os anos de 2000 a 2021. Sugiro que os autores esclareçam essa escolha.

Para o cálculo de anos vividos com incapacidade (YLD), os autores mencionam que foram utilizados dados de expectativa de vida fornecidos pelo estudo sobre Carga Global de Doenças do ano de 2021 (referência 14). Sugiro que os autores mencionem a idade esperada para o óbito que foi considerada para o cálculo de YLD e que são mencionadas no referido estudo (referência 14).

Comentários para a seção de Discussão

Os parágrafos da discussão necessitam ser reorganizados de forma que sigam a ordem de: a) síntese dos achados principais; b) discussão dos achados à luz da literatura; c) apresentação das limitações e pontos fortes; d) implicação dos resultados para o serviço de saúde; e) conclusão.

